# Implementation of pooled saliva tests for universal screening of cCMV infection

**DOI:** 10.1038/s41591-024-02873-3

**Published:** 2024-03-08

**Authors:** Lior Merav, Noa Ofek Shlomai, Esther Oiknine-Djian, Orit Caplan, Ayala Livneh, Tal Sido, Amir Peri, Aviad Shtoyer, Eden Amir, Kerem Ben Meir, Yutti Daitch, Mila Rivkin, Esther Kripper, Irit Fogel, Hadar Horowitz, Sraya Greenberger, Mevaseret Cohen, Miriam Geal-Dor, Oren Gordon, Diana Averbuch, Zivanit Ergaz-Shaltiel, Smadar Eventov Friedman, Dana G. Wolf, Moran Yassour

**Affiliations:** 1 School of Computer Science and Engineering, The Hebrew University of Jerusalem, Jerusalem, Israel; 2 Department of Microbiology and Molecular Genetics, IMRIC, Faculty of Medicine, The Hebrew University of Jerusalem, Jerusalem, Israel; 3 Clinical Virology Unit, Department of Clinical Microbiology and Infectious Diseases, Hadassah Hebrew University Medical Center, Jerusalem, Israel; 4 Department of Neonatology, Hadassah and Hebrew University Medical Center, Jerusalem, Israel; 5 Hebrew University Faculty of Medicine, Jerusalem, Israel; 6 Lautenberg Center for General and Tumor Immunology, Jerusalem, Israel; 7 Computing Department of Laboratories and Institutes, Hadassah Hebrew University Medical Center, Jerusalem, Israel; 8 Speech and Hearing Center, Hadassah-Hebrew University Medical Center, Jerusalem, Israel; 9 Department of Communication Disorders, Hadassah Academic College, Jerusalem, Israel; 10 Pediatric Infectious Diseases, Pediatric Division, Hadassah Hebrew University Medical Center, Jerusalem, Israel

**Keywords:** Population screening, Viral infection

## Abstract

Congenital cytomegalovirus (cCMV) is the most common intrauterine infection, leading to neurodevelopmental disabilities. Universal newborn infant screening of cCMV has been increasingly advocated. In the absence of a high-throughput screening test, which can identify all infected newborn infants, the development of an accurate and efficient testing strategy has remained an ongoing challenge. Here we assessed the implementation of pooled saliva polymerase chain reaction (PCR) tests for universal screening of cCMV, in two hospitals of Jerusalem from April 2022 through April 2023. During the 13-month study period, 15,805 infants (93.6% of all live newborn infants) were screened for cCMV using the pooled approach that has since become our routine screening method. The empirical efficiency of the pooling was six (number of tested newborn infants per test), thereby sparing 83% of the saliva tests. Only a minor 3.05 PCR cycle loss of sensitivity was observed for the pooled testing, in accordance with the theoretical prediction for an eight-sample pool. cCMV was identified in 54 newborn infants, with a birth prevalence of 3.4 per 1,000; 55.6% of infants identified with cCMV were asymptomatic at birth and would not have been otherwise targeted for screening. The study demonstrates the wide feasibility and benefits of pooled saliva testing as an efficient, cost-sparing and sensitive approach for universal screening of cCMV.

## Abstract (web summary)

Building on a pre-existing pooling pipeline for the efficient detection of SARS-CoV-2, the results of a large-scale implementation study show the feasibility and benefits of saliva sample pooling to enhance neonatal screening for congenital cytomegalovirus.

## Main

Congenital cytomegalovirus (cCMV) infection is the most common intrauterine infection, with an average worldwide birth prevalence of 6.4–7 per 1,000 live births, and regional birth prevalence rates ranging between 2 and 13 per 1,000 in different populations^1–9^. Approximately 20% of congenitally infected infants suffer from neurodevelopmental disabilities or sensorineural hearing loss (SNHL), which can be apparent at birth or develop later during childhood^6,9–11^. Most (approximately 90%) congenitally infected newborn infants are asymptomatic at birth; however, 10–15% of initially asymptomatic infants with cCMV will later develop permanent sequelae, with SNHL being the most frequent long-term complication^6,9,10,12^.

Early antiviral treatment with oral valganciclovir for 6 months, when initiated within the first month of life, has been demonstrated to improve hearing and suggested to improve developmental outcomes in children with moderately to severely symptomatic cCMV^6,11,13^. Additionally, screening for cCMV in infancy facilitates timely diagnosis and nonpharmacological management of late-onset SNHL, with notably improved speech and language functions^14–20^.

While the optimal screening strategy of cCMV has remained uncertain, targeted screening of high-risk newborn infants is currently used in many centers, focusing on infants with a failed newborn hearing screen, history of maternal infection or otherwise clinically suspected cCMV^6,8,14,16,21–25^. However, this approach misses most congenitally infected infants who are asymptomatic at birth, yet at risk for late-onset sequelae^7,9,10,16,26–28^. Thus, universal newborn infant screening of cCMV has been increasingly advocated, to allow for early identification of all infected infants^5,7,9,11,14,17,29,30^.

A major consideration in the implementation of universal newborn infant screening for cCMV is the accuracy and feasibility of the screening test. Screening of cCMV using PCR of dried blood spots (DBS) advantageously uses available universal collection infrastructure, but has demonstrated variable sensitivities^11,31–34^. Even with a recently improved average detection sensitivity of 75%, DBS PCR would still miss one-quarter of newborn infants with cCMV^5^, suggesting that further optimization may be required to make the DBS test more robust for universal cCMV screening. In recent years, saliva PCR has been widely used as the reference method for targeted cCMV screening^9,11,35–37^. While saliva PCR testing is highly sensitive, it has been found by us and by others to yield high rates of false positive results (mostly related to peri-partum saliva contamination by CMV DNA from breast milk), therefore requiring routine urine test confirmation of positive saliva results^7,8,21^. This limitation, along with the prospected increased expenses associated with expanding saliva testing to include all newborn infants, limit the suitability of saliva PCR testing in its current form for large-scale universal cCMV screening and call for the development of a more efficient testing strategy.

A promising approach to increase testing throughput while saving resources is sample pooling. In the simplest Dorfman pooling method, each sample is assigned to a single pool, the pools contain equal numbers of samples, all samples in negative result pools are declared negative and samples are retested individually only if the pool’s test result is positive^38^. Because the expected efficiency of pooling depends on the prevalence rate of the measured parameter, the low overall birth prevalence of cCMV makes it an ideal candidate for pooled screening. In addition, as cCMV is characterized by a high viral load in saliva, the loss of sensitivity on sample dilution by pooling should not be a major theoretical concern. In this regard, a few recent proof-of-concept studies suggested the sensitivity of pooling for cCMV testing, yet they included only a few hundred neonates^39–42^. A study in 2023 described the use of pooled saliva testing in 7,033 newborn infants screened for cCMV over a 2-year period^43^. Yet, this study presented limited results, merely describing the feasibility of pooling, with no additional analyses (that is, sensitivity, efficacy and operation). Recently, at the onset of the coronavirus disease 2019 pandemic, we successfully implemented large-scale Dorfman sample pooling for the efficient detection of severe acute respiratory syndrome coronavirus 2 (SARS-CoV-2)^44,45^.

In this study, building on our preexisting pooling pipeline, we validated and applied large-scale pooling of saliva PCR testing for universal screening of cCMV, replacing our former targeted screening strategy. Addressing the three key considerations pertaining to this newly established cCMV screening approach, namely, sensitivity, efficiency and feasibility, we describe the lessons derived from the universal cCMV screening of more than 15,000 newborn infants over a 13-month period. We carried out a theoretical analysis of all cCMV tests performed in 2014–2021 (before the implementation of pooling) to evaluate and predict the sensitivity of the pooling method and its ability to filter false positive results. After the initial experimental validation, we further performed ongoing prospective validation of our pooling strategy by retesting of samples that belonged to negative pools (approximately 5% of the negative pools), establishing the high negative predictive value (NPV) of our pooling approach. We compared values from two CMV genes used for detection (*IE* and *gB*), thereby increasing our sensitivity, and measured the sensitivity over time (comparing the cycle threshold (Ct) of pools with the Ct of individual samples). Finally, we calculated the empirical real-world efficiency of our approach. Our extensive analysis revealed the high empirical efficiency of pooled saliva testing, sparing approximately 83% of the tests compared with individual testing, with a clinically insignificant loss of sensitivity. Together with the high operational feasibility and acceptance rate, our findings support the widespread implementation of pooled saliva testing for universal screening of cCMV.

## Results

**Fig. 1:**
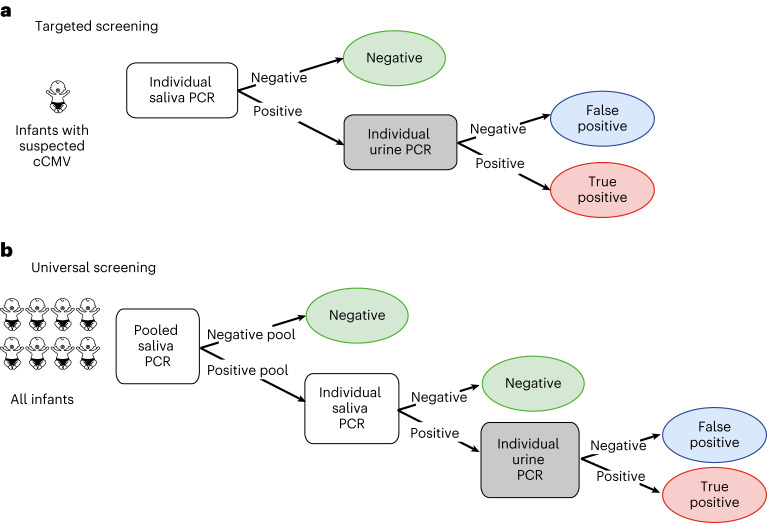
Schematic presentation of neonatal cCMV screening algorithms at the Hadassah Medical Center. **a**, Targeted screening algorithm of suspected neonates by conventional individual saliva RT–PCR with confirmatory urine testing (used in 2014–2022). **b**, Universal screening algorithm, applying the newly implemented saliva sample pooling (beginning in April 2022).

In the past, infants born at Hadassah Medical Center were subjected to targeted screening for cCMV (Fig. 1a)^21^. Infants with a positive saliva real-time (RT) PCR who test positive for CMV in urine are considered positive for cCMV infection (true positive saliva detection), whereas infants with positive saliva RT–PCR who test negative in urine are considered to have false positive saliva detection (Fig. 1a). Using this approach, we found that a considerable proportion (approximately 55%) of positive saliva samples, mainly those with low viral load, were false positive^21^.

In this study, we developed a new pooling setup for universal screening of cCMV, using lessons derived from our large-scale sample pooling to detect SARS-CoV-2 (refs. ^44,45^). According to this setup, the saliva samples of all parent-consented newborn infants were tested (including DNA extraction and RT–PCR tests) in pools (Fig. 1b). If the pool RT–PCR was negative, all samples within the pool were defined as negative. If the pool tested positive, it was ‘opened’ and all saliva samples within the pool were retested individually. Once a CMV-positive saliva specimen was identified within a pool, a urine confirmatory test was performed and cCMV (true positive saliva detection) versus false positive saliva detection were defined (Fig. 1b).

**Extended Data Fig. 1:**
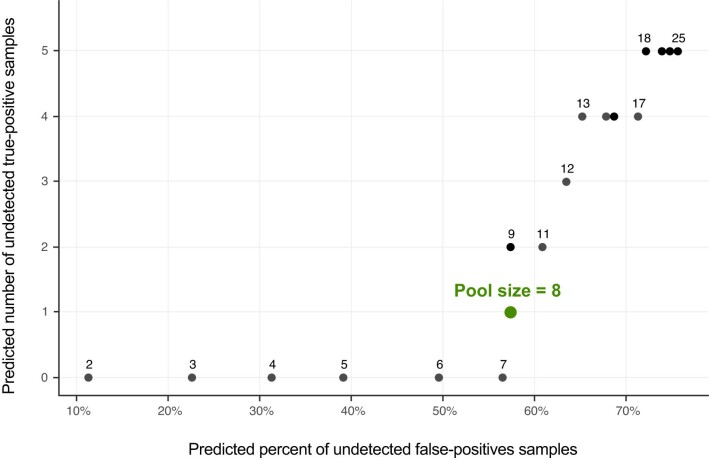
The calculated effect of saliva-sample pool size on the accuracy of cCMV detection. The dots represent the different pool sizes, as indicated by the numbers above the dots. The parameters shown were calculated for each pool size, and include the number of true-positive cCMV cases that would have become undetected by using each pool size (Y axis), and the percent of false-positive saliva detection cases that would have become undetected by using each pool size (X axis). These data were retrospectively calculated for all (8,643) newborns who underwent targeted screening for cCMV in 2014–2021.

Along with the improved testing capacity, a key requirement of pooled sample testing is to retain high clinical accuracy with sufficient sensitivity. To determine the optimal pool size, we first assessed the expected clinical accuracy of different pool sizes for cCMV detection. To this end, we looked back at the data of all newborn infants who underwent testing for cCMV during 2014–2021 and calculated the potential effect of different saliva sample pool sizes on (1) the number of true positive cCMV cases that would have been missed by sample pooling and (2) the percentage of false positive saliva detection cases that would have been spared by sample pooling. These parameters were theoretically calculated based on the expected relative increase of the pooled saliva RT–PCR Ct value (Extended Data Fig. 1). We found that a pool size of *n* = 8 would maintain a sensitivity of 99.5% (missing 1 of 188 of the true positive cCMV cases). The single potentially missed cCMV case was a congenitally infected newborn infant with very low viral loads in the saliva (325 DNA copies per ml; 28.5 IU ml^−1^) and urine (1,190 copies per ml; 116 IU ml^−1^), associated with late-gestation maternal infection, who remained asymptomatic by age 7 years. Importantly, we also showed that an eight-sample pooling approach would have filtered 57% (66 of 115) of the false positive saliva tests during this period (Extended Data Fig. 1).

After experimental validation of the eight-sample pooling (Methods), universal screening was gradually introduced during a pilot period (January–March 2022), applied in a single nursery department, with targeted screening continued in all other nursery departments. During this 3-month period, all saliva samples arriving at the laboratory were tested in parallel using sample pooling and conventional individual sample testing, as a real-world experimental validation. Of 196 pools tested during this period, eight were positive and 188 were negative (Supplementary Data 1). When tested individually, all 1,414 samples in the negative pools were negative, with a NPV of 100%. Of the eight positive pools, seven (88%) contained a positive saliva sample when opened and tested individually. All of the individually tested positive samples were also found in the pooled setting, again confirming the sensitivity of our pooling method.

**Fig. 2:**
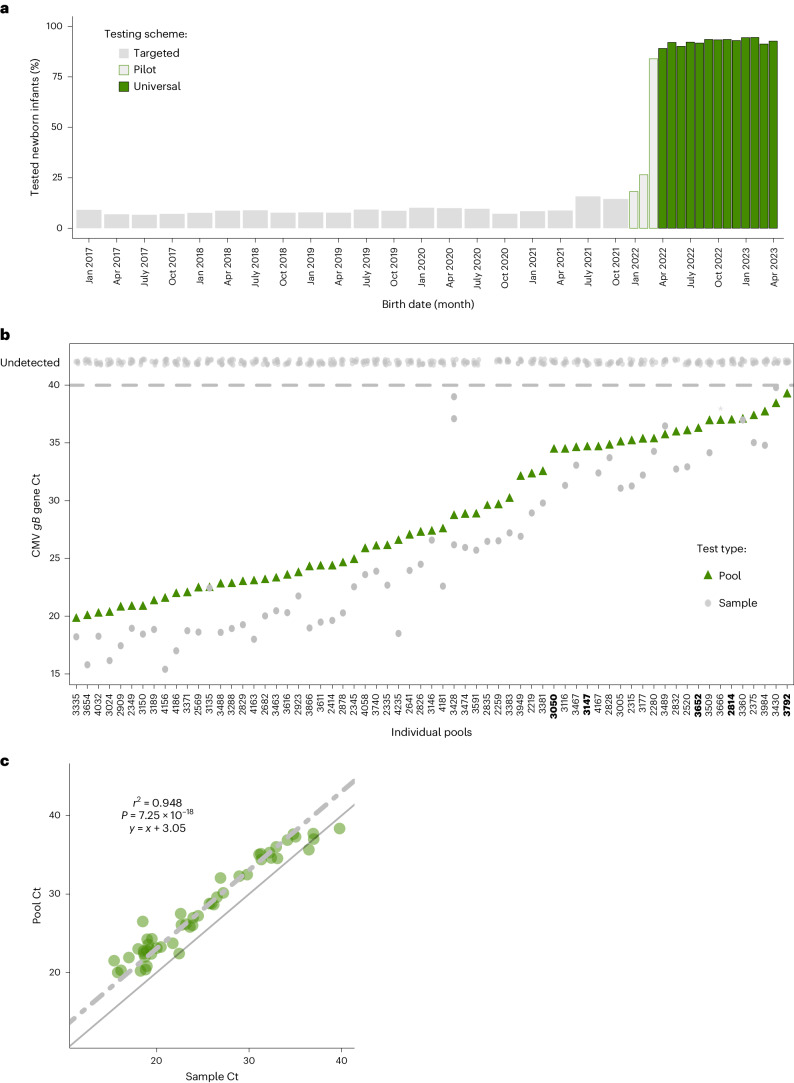
Overall performance of pooled saliva testing for universal screening of cCMV. **a**, Percentage of live newborn infants tested using (individual saliva sample testing-based) targeted screening (Targeted, gray), during the transition from individual saliva sample targeted screening to universal pooled saliva screening (Pilot, light gray with green outline) and since the routine implementation of universal pooled testing (Universal, green, beginning in April 2022). The wide and narrow bars represent 3-month and 1-month periods, respectively. **b**, Comparison of the CMV *gB* Ct values of the pool (green triangle) and the individual samples within the pool (gray circles) for the 65 positive pools that had a *gB* Ct value (Extended Data Fig. 2). Samples with undetected Ct values are shown at the top. Bold pool IDs represent pools that did not have any positive sample after individual saliva PCR testing (for *gB* or *IE*). Pool 3666 had a single positive sample that was detected only using the *IE* gene PCR, marked by a single asterisk. **c**, Linear regression plot showing the relationship between the Ct values of 47 positive eight-sample pools (*y* axis) and the Ct values of the corresponding single positive samples in the pools (*x* axis). Each green dot represents a positive pool and its corresponding individual positive sample. Linear regression with a predetermined slope of 1 is marked by the upper dashed gray line; *y* = *x* is marked in a solid gray line. The regression formula and statistical measure appear in the text above the fit (two-tailed *t*-test, calculated using the lm function in R; Methods).

After the pilot period, universal neonatal cCMV screening using saliva sample pooling was routinely implemented at the two hospitals of Hadassah Medical Center beginning in April 2022 (Fig. 2a). During the 13-month period between April 2022 and April 2023, 15,805 neonates, representing 93.6% of all 16,884 live newborn infants, were screened for cCMV using 1,990 pools. Of these, 15,273 neonates (90.5% of all live newborn infants and 96.6% of the screened newborn infants) were screened using pooled saliva. An additional 532 neonates were screened individually because of daily logistic considerations (for example, to avoid testing delay at the end of the working day when not all eight-sample pools could be filled; Supplementary Data 1). This high screening rate represented a drastic increase compared to the low proportions of infants screened for cCMV during the targeted screening period (with 10.1% average monthly percentage of targeted screening calculated for 2016–2021; Fig. 2a). The rate of cCMV screening was similar to that of other routine newborn screening performed at Hadassah Medical Center (such as universal metabolic screening, hearing screen and oxygen saturation screening for the detection of congenital cyanotic heart diseases).

Overall, cCMV was identified in 54 of 15,805 screened newborn infants, with a birth prevalence of 3.4 per 1,000 (95% confidence interval (CI) = 2.6%–4.3%). Importantly, 30 (55.6%) infants with cCMV were tested and identified only because of the universal screening and would not have been tested (and therefore missed) by targeted screening. Of these 30 infants, one had intracranial involvement, revealed by ultrasound, leading to early valgancyclovir treatment.

**Extended Data Fig. 2:**
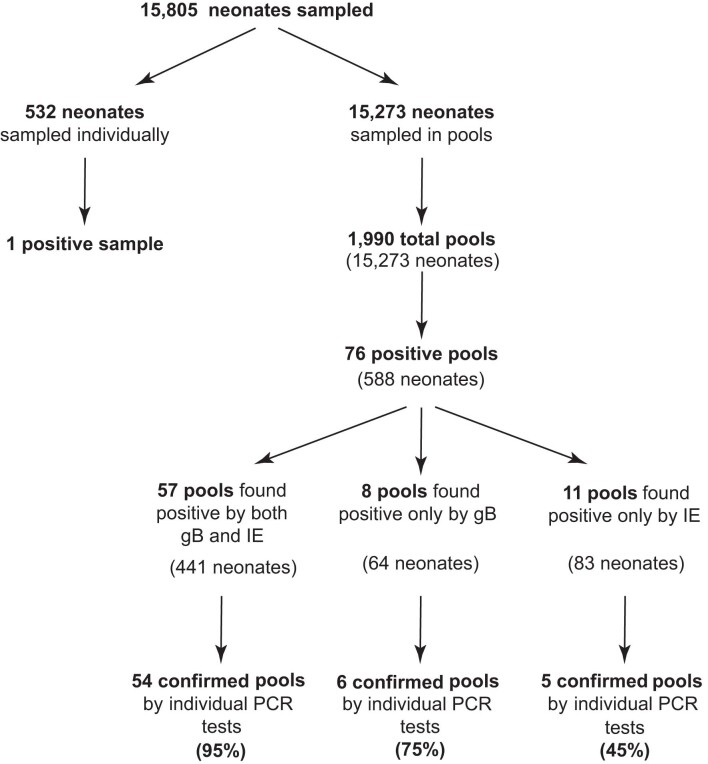
The overall number of samples and neonates tested in April 2022-April 2023. Samples were either pooled (right) or tested individually, due to daily logistic considerations (left). Positive pools were defined as those that have a positive *gB* and/or *IE* gene PCR Ct value. All individual samples in positive pools were subsequently tested by saliva PCR.

Of the 1,990 tested pools, only 76 (3.82%) tested positive for *IE* or *gB* genes (Fig. 2b, Extended Data Fig. 2 and Supplementary Data 1). Most pools contained eight saliva samples each, except for infrequent occasions when fewer samples (5–7) were pooled, or rarely, tested individually. In accordance with the testing algorithm (Fig. 1b), all samples in the positive pools were retested individually. Most (85.5%) positive pools contained at least one positive sample (Fig. 2b). A smaller fraction of the positive pools (14.5%) did not yield any positive sample when their samples were retested individually (Supplementary Data 1). This low percentage of false positive pools (all of which had Ct values above 34, or detected only using *IE* or *gB* gene RT–PCR) reflected our extra caution with ‘permissive’ pool opening at any signal, taken to maintain the sensitivity of pooled sample testing.

The maintenance of sensitivity is a major concern in the pooled testing setup. We evaluated the empirical sensitivity of our routine eight-sample pooling approach by comparing the pool Ct value with the individual sample Ct value for all the 47 eight-sample pools that contained (each) a single positive sample. This comparison, using linear regression analysis with a slope of 1, revealed a 3.05 Ct increase for the pool relative to the individual positive sample (Fig. 2c). Notably, this empirical finding was in agreement with the theoretical prediction of a 3-Ct increase on eight-sample pooling (log_2_ of the dilution factor). To further control for potential false negative results of the pooling, 92 negative pools (constituting 4.8% of all the negative pools and containing a total of 736 samples) were randomly opened and their pooled samples tested individually. These ongoing quality control tests did not identify any true positive cCMV infection (one false positive saliva sample with Ct = 37 for the *IE* gene and negative for the *gB* gene was identified). Together, the combined monitoring measures support the ongoing empirical sensitivity of our routine large-scale pooled testing.

A major advantage of the pooling approach is efficiency, defined according to the number of samples (infants) tested using a single RT–PCR reaction. Assessing our empirical efficiency, we found that during the period between April 2022 and April 2023, 15,273 saliva samples were tested in 1,990 pools. For this purpose, only 2,578 RT–PCR reactions were performed, with an efficiency of 5.92 samples per RT–PCR reaction. For comparison, during the equivalent time in 2021–2022, 1,275 saliva samples were tested using 1,275 RT–PCR reactions. Thus, with an efficiency of approximately six samples per PCR reaction, pooled testing allowed us to use twice as many PCR reactions to test 12-fold more samples.

## Discussion

The development of an efficient testing strategy to enhance universal screening of cCMV is recognized as a high priority goal^9,11^. In this study, we report on the implementation of eight-sample pooling of saliva samples for universal screening of cCMV. Data analysis of more than 15,000 pooled samples tested over a 13-month period revealed the high empirical efficiency and feasibility of pooled saliva testing, outweighing a negligible potential loss of sensitivity.

Our pooled testing strategy allowed the universal screening of nearly 94% of live newborn infants with an empirical efficiency of approximately six, thereby sparing approximately 83% of saliva tests. Given the low prevalence rate of cCMV, the vast majority (96.18%) of the pools were negative, obviating the need for individual sample (re)testing. Beyond the reduction of saliva tests per se, an important secondary beneficial effect of the pooling approach was the significant reduction in the rate of false positive low viral load saliva results (calculated to be 57%), which were filtered by the slight decrease in the pooled assay sensitivity compared to individual saliva testing (Extended Data Fig. 1). Thus, while enabling us to drastically increase the throughput of the first saliva screening step, the pooled saliva assay also reduced the second (often unnecessary and inconvenient) newborn urine collection and confirmation step.

When evaluating the clinical implementation of pooled saliva sample testing, the potential loss of sensitivity upon dilution, affecting mainly samples with a low viral load, should be considered. However, in the case of cCMV, which is characterized by high viral load in saliva, this concern can be waived. This may not be an ideal approach for other infections with potential low viral loads, where additional approaches can be considered^46^. Based on the distribution of the saliva viral loads among our previously individually tested cCMV cases, we predicted a detection sensitivity of 99.5% for the eight-sample pooling (Extended Data Fig. 1). In reality, our empirical sensitivity, analyzed during the routine implementation of pooled saliva testing, showed an approximate 3-Ct loss, in accordance with the theoretical prediction for eight-sample pools (Fig. 2). We further monitored the sensitivity of pooled testing after our initial experimental validation, by assessing the NPV of all negative pools during the 3-month pilot, and of randomly selected approximately 5% of the negative pools during the 13-month routine implementation period. Only a single sample was positive (false positive, with Ct = 37) suggesting an NPV of 100%. Thus, we believe that the loss of sensitivity on eight-sample pooling is clinically insignificant with respect to cCMV detection. However, large-scale implementation of pooled screening at multiple centers and with larger populations may miss more than single cases. Additionally, like other screening tests, individual infants may need to be tested if clinical suspicion remains for a congenital CMV infection.

A critical consideration with regard to universal screening via sample pooling, refers to the feasibility and logistics and simplicity of operation. A recent study suggested the feasibility of pooling; however, this study included a smaller number of newborn infants (less than half the number of newborn infants screened in the present study), screened over a longer period (2 years), and did not relate to operational considerations^43^. In this study, using our preexistent pooling pipeline, we were able to rapidly incorporate neonatal saliva pooling into routine clinical practice. This pipeline, which we believe can be easily implemented in diagnostic laboratories that serve similar to larger-size maternity units, includes automation of sample handling and processing by using an easy robotic setup and a dedicated software (ref. ^45^ and Methods), with rapid notification of the results. Importantly, at our neonatal sample rate, where pools could be filled within a day, confirmation and clinical evaluation of most cCMV cases took place within the first 2–3 days of life, while still in the newborn nursery. Results may not arrive while still in the normal newborn nursery at many sites and countries; in these cases, the parents should be contacted for further confirmation, guidance and follow-up. Beyond the operational aspects, one should bear in mind that sample pooling requires a change in the frame of mind and approach to sample handling by technical staff, who are generally trained to meticulously preserve the integrity of individual samples. The concern of sample contamination while introducing the pooling pipeline was minimized by automatic aliquoting with storing and tracing of each individual sample within the pool. It was well accepted and rapidly integrated into staff training.

Our routine implementation of cCMV universal screening in the two hospitals of the Hadassah Medical Center, serving the general population of Jerusalem, yields important interim epidemiological and clinical observations. Overall, 54 out of 15,805 screened infants were identified with cCMV using urine confirmatory testing, with a birth prevalence of 3.4 per 1,000 (95% CI = 2.6–4.3 per 1,000). This observed prevalence is in the range of that reported in Western European countries in similar-scale studies^7,8^. While it is somewhat lower than the 4–7 per 1,000 birth prevalence previously reported in two small studies^39,47^ and in one similar-scale study^48^ in Israel, the findings highlight the importance of ongoing large-scale screening in defining the true population prevalence of cCMV. In this regard, it is noteworthy that the population of Jerusalem uniquely consists of segregated communities characterized by different maternal fertility and seroprevalence rates^49^. As cCMV prevalence varies considerably according to ethnic grouping, ethnicity and maternal seroprevalence^1,4,9,12,50^, it will be important to further assess the prevalence of cCMV in distinct subpopulations.

Lastly, the high rate of our cCMV screening, nearing 94% of all newborn infants, was similar to that of other routine newborn screening in our center, thereby reflecting high parental acceptance of cCMV screening. This finding, which is in line with recent studies reporting high rates of parental consent, mitigates earlier concerns regarding undue parental anxiety and reluctance to cCMV screening^5,7,51,52^.

More than half of the congenitally infected infants identified by the newly implemented universal screening (30 of 54; 55.6%) would have been ‘missed’ by our previous targeted screening approach. This finding is in agreement with the general recognition that in the absence of universal screening, most cCMV cases remain undiagnosed^7,9,11^. Of the 30 well-appearing infants who were identified and consequently further evaluated because of universal screening, one had intracranial involvement, leading to early administration of antiviral treatment. Studies are currently underway in our center to examine the rate and risk factors for early and late cCMV-related complications in universally screened infants.

This study has several limitations. First, we were not able to fully estimate the false negative rate of pooled testing during routine implementation because not all samples included in the pools were tested individually. However, the ongoing individual retesting of samples included in approximately 5% of the negative pools, along with the continued surveillance of the empirical loss of sensitivity, support the sustained sensitivity of our pooling approach. Second, while the high empirical efficiency should imply significant assay cost reduction, the study did not address the cost effectiveness of our pooled sample universal cCMV screening approach, which should be assessed in future studies. As the study population is limited to the two hospitals of Hadassah Medical Center in Jerusalem, our estimate of the overall prevalence of cCMV over a 13-month period may not be representative of the entire population in Israel. Nonetheless, the two hospitals cover the general population of Jerusalem; the large cohort size together with the heterogeneity of our population argue for the generalizability of our findings.

In summary, the data presented in this study project on the wide feasibility and benefits of saliva sample pooling to enhance universal neonatal screening for cCMV. The pooling setup described can be easily integrated in any medium-to-large medical laboratory with an expected sixfold efficiency in populations with similar prevalence rates. Beyond the direct clinical implications toward early diagnosis, monitoring and treatment of cCMV, data derived from the implemented universal screening will serve to define the burden, risk factors and clinical outcomes of cCMV over time and to increase awareness of this underrecognized congenital infection.

## Methods

### Study design

This prospective, population-based cohort study was conducted at the two hospitals of Hadassah Medical Center (including seven newborn nurseries, two intermediate care neonatal units and two neonatal intensive care units) from April 2022 through April 2023. After prior design and validation of pooled saliva RT–PCR testing for the detection of cCMV (see the experimental validation of the pooling scheme below and ʻResultsʼ), and a 3-month pilot implementation period (January–March 2022; ʻResultsʼ), all newborn infants whose parents provided written informed consent were screened for cCMV using pooled saliva RT–PCR testing as part of a routinely implemented newborn screening policy. Infants with positive saliva tests (identified by the initial pooled testing with subsequent retesting of all samples in the positive pool; see below) had confirmatory urine testing (Fig. 1). Urine samples were tested for CMV using RT–PCR. All saliva specimens and most urine specimens were collected during the first 1–3 days of life. All specimens were collected no later than 10 days after birth. The study was approved by the Hadassah Medical Center institutional review board (no. 0272-22-HMO).

### Collection and testing of saliva and urine samples

Saliva specimens (obtained by swabbing the infant’s buccal mucosa) were immediately placed in closed tubes containing 3 ml Universal Transport Medium (COPAN). Urine specimens were collected in sterile bags. All specimens were immediately transported to the Hadassah Medical Center Clinical Virology laboratory. CMV PCR was run daily (5 days per week). Samples were stored at 4 °C until tested on the same day or up to 72 h after collection.

### DNA extraction and RT–PCR detection for individual tests

DNA was extracted from saliva or urine samples (200 μl of Universal Transport Medium-immersed oral swab, or urine sample, yielding 50 μl of eluted DNA) using MagnaPure (Roche Life Sciences). RT–PCR was performed using 5 μl of the eluted DNA in a 25-μl reaction, with the use of primers and probes derived from the CMV glycoprotein B (for quantitation) and immediate early 1 (*IE1*) genes along with a housekeeping gene (human *ERV3*), using the TaqMan Fast Advanced Master Mix (Applied Biosystems) on the QuantStudio 5 Real-Time PCR instrument (Applied Biosystems).

The PCR primer sequences were: *gB,* forward 5′-TGGGCGAGGACAACGAA-3′, reverse 5′-TGAGGCTGGGAAGCTGACAT-3′; *IE1,* forward 5′-TCCCGCTTATCCTCRGGTACA-3′, reverse 5′-TGAGCCTTTCGAGGASATGAA-3′; *ERV3*, forward 5′-CATGGGAAGCAAGGGAACTAATG-3′, reverse 5′- CCCAGCGAGCAATACAGAATTT.

The PCR probe sequences were: *gB* FAM, 5′-TGGGCAACCACCGCACTGAGG-3′; *IE1* FAM/VIC 5′-TCTCATACATGCTCTGCATAGTTAGCCCAATACA-3′; *ERV3* cyanine5, 5′-TCTTCCCTCGAACCTGCACCATCAAT-3′.

The assay demonstrated a linear quantitation over a 6-log range with a sensitivity of 50–100 copies per ml. A viral DNA load of 50 copies per ml or higher was considered positive.

### Pooled testing of saliva samples

Individual samples were pooled on a Tecan Robot. For the 1:8 pool, equal volumes of eight samples were pooled to a final volume of 3.2 ml. Sometimes, pools with fewer samples (5–7) were used or rarely tested individually (for example, to avoid testing delay at the end of the working day in cases that not all eight-sample pools could be filled). DNA was extracted (from 200 μl of the pooled sample, yielding 50 μl elution) and subjected to RT–PCR as described above. A negative result implied that all samples in the pool were negative, while a positive result implied that at least one sample in the pool was positive. The samples of each pool that tested positive (any Ct of 40 or less) were subsequently individually tested (Fig. 1). All steps that could affect repeatability, reproducibility, sensitivity, specificity and trueness were evaluated on a regular basis.

The IT system supported the pooling process, allowing sample location, tracking, assignment for retesting, reporting and data analysis for the different stages of pooling^44^.

### Experimental validation of the pooling scheme

To validate our pooling strategy, we tested assay sensitivity by mixing one positive sample with seven negative samples. The sensitivity of pooling was evaluated across a range of viral loads by diluting a quantitative standard with decreasing viral loads (5,000, 2,500, 1,000, 500 and 250 viral DNA copies per ml, corresponding to the detected Ct range of 33–37), mixing each dilution with seven negative samples. All these dilutions were detected and quantitated in the pooled testing. We further tested the pooling of 160 saliva samples into 20 pools of eight samples each, combining seven negative saliva samples and one positive sample (with a Ct range of 20–37) and also tested in parallel each sample individually. Each of the 17 pools that contained one positive sample was positive for the CMV *gB* or *IE1* gene; all three pools that contained only negative samples were negative. This finding revealed highly accurate results of the pooling, with no loss of diagnostic assay sensitivity.

### Statistical analysis

Analysis was performed as in our evaluation of the SARS-CoV-2 pooling scheme^45^. Pooling efficiency was calculated as the number of samples that could be tested per single PCR reaction.

Data were analyzed with R, using common packages such as dplyr (v.1.1.4) and stats (v.3.6.2), and plotted with ggplot2 (v.3.4.4). Linear regression of pool versus sample Ct values was done using the lm package in R, constraining the slope of the regression to 1.

### Pool Ct versus sample Ct calculation

The PCR reaction roughly multiplies the amount of the targeted DNA in each cycle of operation. Because of this exponential growth, a pool of size *n* with a single positive sample should have a Ct that is log_2_(*n*) cycles greater than the positive sample’s Ct. For example, when the pool size is eight, this will result in a three-cycle addition. The regression *r*^2^ and *P* values were calculated from the model inferred by the fit returned from the lm function.

### Reporting summary

Further information on research design is available in the Nature Portfolio Reporting Summary linked to this article.

## Online content

Any methods, additional references, Nature Portfolio reporting summaries, source data, extended data, supplementary information, acknowledgements, peer review information; details of author contributions and competing interests; and statements of data and code availability are available at 10.1038/s41591-024-02873-3.

### Supplementary information

Reporting Summary

Supplementary Data 1 Full data of the pooled and nonpooled saliva tests. For each sample, we indicate its pool number (if it was pooled), the Ct values for the pool and for the sample (if that pool was opened), and the final result when incorporating the urine test.

## Data Availability

All raw data of the PCR values for all samples and pools are shown in Supplementary Data 1.

## Code availability

No custom algorithmic code was used to analyze the data in this manuscript.
