# Supplementary material for: Implementation of pooled saliva tests for universal screening of cCMV infection
Source: Nat Med. 2024 Mar 8;30(4):1111–7. doi: 10.1038/s41591-024-02873-3 (PMC11031397; doi:10.1038/s41591-024-02873-3)
Supplement: Supplementary file 1 — Reporting Summary [file 41591_2024_2873_MOESM1_ESM.pdf]

nature portfolio

Corresponding author(s): Yassour Moran

Last updated by author(s): Dec 29, 2023

Reporting Summary

Nature Portfolio wishes to improve the reproducibility of the work that we publish. This form provides structure for consistency and transparency in reporting. For further information on Nature Portfolio policies, see our Editorial Policies and the Editorial Policy Checklist.

Statistics

For all statistical analyses, confirm that the following items are present in the figure legend, table legend, main text, or Methods section.

n/a	Confirmed	
☐	☒	The exact sample size (*n*) for each experimental group/condition, given as a discrete number and unit of measurement
☒	☐	A statement on whether measurements were taken from distinct samples or whether the same sample was measured repeatedly
☐	☒	The statistical test(s) used AND whether they are one- or two-sided<br>*Only common tests should be described solely by name; describe more complex techniques in the Methods section.*
☒	☐	A description of all covariates tested
☐	☒	A description of any assumptions or corrections, such as tests of normality and adjustment for multiple comparisons
☒	☐	A full description of the statistical parameters including central tendency (e.g. means) or other basic estimates (e.g. regression coefficient) AND variation (e.g. standard deviation) or associated estimates of uncertainty (e.g. confidence intervals)
☐	☒	For null hypothesis testing, the test statistic (e.g. *F*, *t*, *r*) with confidence intervals, effect sizes, degrees of freedom and *P* value noted<br>*Give P values as exact values whenever suitable.*
☒	☐	For Bayesian analysis, information on the choice of priors and Markov chain Monte Carlo settings
☒	☐	For hierarchical and complex designs, identification of the appropriate level for tests and full reporting of outcomes
☒	☐	Estimates of effect sizes (e.g. Cohen's *d*, Pearson's *r*), indicating how they were calculated

Our web collection on statistics for biologists contains articles on many of the points above.

Software and code

Policy information about availability of computer code

Data collection	Data were collected using the centralized EMR system at Hadassah Medical Center
Data analysis	Data were analyzed with R, using common packages such as dplyr (version 1.1.4) and stats (version 3.6.2), and plotted with ggplot2 (version 3.4.4). Linear regression was done using the lm package in R.

For manuscripts utilizing custom algorithms or software that are central to the research but not yet described in published literature, software must be made available to editors and reviewers. We strongly encourage code deposition in a community repository (e.g. GitHub). See the Nature Portfolio guidelines for submitting code & software for further information.

Data

Policy information about availability of data

All manuscripts must include a data availability statement. This statement should provide the following information, where applicable:

- Accession codes, unique identifiers, or web links for publicly available datasets
- A description of any restrictions on data availability
- For clinical datasets or third party data, please ensure that the statement adheres to our policy

All data are available in the Supplementary Data

Human research participants

Policy information about studies involving human research participants and Sex and Gender in Research.

Reporting on sex and gender	This information has not been collected in this study
Population characteristics	This information has not been collected in this study
Recruitment	All newborns whose parents provided written informed consent were screened for cCMV by pooled saliva RT-PCR testing as part of a routinely implemented newborn screening policy. At admission to the labor & delivery department at Hadassah Medical Center, parents are handed a consent form for all provided newborn screening tests, with explanations for each test. To the best of our knowledge, there is no bias in the consent process. The high consent rate (nearing 94%) spans multiple cultural groups and ethnic background.
Ethics oversight	The study was approved by the Hadassah Medical Center Institutional Review Board (#0272-22-HMO).

Note that full information on the approval of the study protocol must also be provided in the manuscript.

Field-specific reporting

Please select the one below that is the best fit for your research. If you are not sure, read the appropriate sections before making your selection.

☒ Life sciences ☐ Behavioural & social sciences ☐ Ecological, evolutionary & environmental sciences

For a reference copy of the document with all sections, see nature.com/documents/nr-reporting-summary-flat.pdf

Life sciences study design

All studies must disclose on these points even when the disclosure is negative.

Sample size	We encouraged all families to test their newborns, and we had nearly 94% compliance. We collected the information for 13 months, and this is the total number of tested individuals we report. This period of time was representative of an average year with respect to birth rate at our center.
Data exclusions	We removed CMV testing of infants that were tested at >10 days of age.
Replication	This is a real-world study, where data were collected as infant were born, replication is not aplicable to this study
Randomization	We randomly chose 4.8% of negative pools for re-testing as individual samples to verify ongoing sensitivity of the assay.
Blinding	All investigators of this study were blinding to the group allocation of newborns. When analyzing the data we did not know anything about the outcome of the infants.

Reporting for specific materials, systems and methods

We require information from authors about some types of materials, experimental systems and methods used in many studies. Here, indicate whether each material, system or method listed is relevant to your study. If you are not sure if a list item applies to your research, read the appropriate section before selecting a response.

Materials & experimental systems

n/a	Involved in the study	
☒	☐	Antibodies
☒	☐	Eukaryotic cell lines
☒	☐	Palaeontology and archaeology
☒	☐	Animals and other organisms
☒	☐	Clinical data
☒	☐	Dual use research of concern

Methods

n/a	Involved in the study	
☒	☐	ChIP-seq
☒	☐	Flow cytometry
☒	☐	MRI-based neuroimaging
